# Expression of Fibroblast Activation Protein Is Enriched in Neuroendocrine Prostate Cancer and Predicts Worse Survival

**DOI:** 10.3390/genes13010135

**Published:** 2022-01-13

**Authors:** Panagiotis J. Vlachostergios, Athanasios Karathanasis, Vassilios Tzortzis

**Affiliations:** 1 Division of Hematology and Medical Oncology, Department of Medicine, Weill Cornell Medicine, New York, NY 10065, USA; 2 Department of Urology, University of Thessaly School of Health Sciences Faculty of Medicine, University Hospital of Larissa, 41100 Larissa, Greece; karathanasis.urology@gmail.com

**Keywords:** fibroblast activating protein, neuroendocrine differentiation, prostate cancer, castration-resistant, androgen receptor

## Abstract

Background: Advanced prostate cancer (PC) may accumulate genomic alterations that hallmark lineage plasticity and transdifferentiation to a neuroendocrine (NE) phenotype. Fibroblast activation protein (FAP) is a key player in epithelial-to-mesenchymal transition (EMT). However, its clinical value and role in NE differentiation in advanced PC has not been fully investigated. Methods: Two hundred and eight patients from a multicenter, prospective cohort of patients with metastatic castration-resistant prostate cancer (CRPC) with available RNA sequencing data were analyzed for tumor *FAP* mRNA expression, and its association with overall survival (OS) and NE tumor features was investigated. Results: Twenty-one patients (10%) were found to have high *FAP* mRNA expression. Compared to the rest, this subset had a proportionally higher exposure to taxanes and AR signaling inhibitors (abiraterone or enzalutamide) and was characterized by active NE signaling, evidenced by high NEPC- and low AR-gene expression scores. These patients with high tumor mRNA *FAP* expression had a more aggressive clinical course and significantly shorter survival (12 months) compared to those without altered *FAP* expression (28 months, log-rank *p* = 0.016). Conclusions: *FAP* expression may serve as a valuable NE marker indicating a worse prognosis in patients with metastatic CRPC.

## 1. Introduction

Cancer-associated fibroblasts (CAFs) are an essential component of tumor stroma, with direct involvement in cancer progression via interactions with other cell types within the tumor microenvironment [1]. A key mediator of these interactions is fibroblast activation protein (FAP).

FAP is a transmembrane protease directly implicated in epithelial-mesenchymal transition (EMT) of various tumors, including in the lungs, breast, colorectal, gastric, pancreatic, hepatocellular, head and neck, and skin [2,3,4,5,6,7,8,9]. FAP expression is associated with aggressive tumor features and clinical course including progression and metastasis [2,5,7].

Prostate cancer (PC) represents a disease model for studying EMT, particularly at later stages when there is transition to a neuroendocrine (NE) phenotype under the effect of newer androgen receptor targeted agents (ARTA), such as abiraterone and enzalutamide [10,11]. Castration-resistant NEPC is a distinct clinicopathological and molecular entity compared the typical CRPC adenocarcinoma, with worse prognosis and poor response to systemic therapies [12,13,14]. Therefore, identifying targetable surrogate markers of transition to this NE phenotype is key to overcome resistance and improve outcomes in this subset of patients.

In this study, we assessed the transcriptional expression of *FAP* and its prognostic relevance in patients with metastatic CRPC.

## 2. Materials and Methods

A publicly available database, cBioportal for Cancer Genomics (www.cbioportal.org accessed on 6 January 2022), was used to query RNA sequencing data for *FAP* mRNA expression in a prospective multicenter cohort of 444 tumor samples from 429 patients with mCRPC [15]. Gene expression as fragments per kilobase of exon per million fragments mapped (FPKMs) was determined using featureCounts against protein-coding genes from the Gencode v26 reference [15]. The NEPC score (calculated based on the expression levels of 70 genes) and the AR score (calculated based on the expression levels of 30 genes) were computed by the Pearson’s correlation coefficient between the log2-transformed FPKM values of each score’s gene list and a reference gene expression vector, as previously described [12]. The Cancer Cell Line Encyclopedia (CCLE) database [16] was used to query various primary cell lines for *FAP* mRNA expression.

The Kaplan–Meier method was used to assess the association between high and unaltered *FAP* mRNA expression with overall survival (OS), using a threshold z-score of ≥1.0 in the mCRPC cohort and in two additional validation cohorts [17,18]. OS was measured from the date of biopsy to time of death or last follow-up. The Chi-squared test was used to compare clinical and pathological characteristics and the Wilcoxon test was used to compare NEPC and AR signaling scores between subgroups with high vs. unaltered FAP mRNA expression. Multiple hypothesis test correction was applied using the Benjamini–Hochberg method. *p* and *q* values of <0.05 were considered significant for all analyses.

## 3. Results

**Figure 1:**
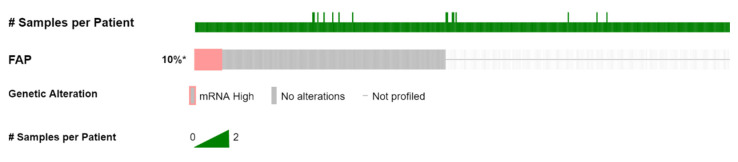
Oncoplot of *FAP* mRNA expression in patients with mCRPC. #: number, *: % of profiled.

**Figure 2:**
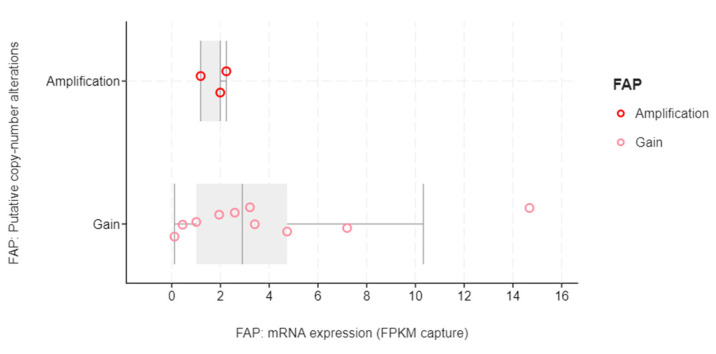
Copy-number alterations associated with high *FAP* mRNA expression in mCRPC patients.

Two hundred and eight patients/samples with available RNA sequencing data out of the entire mCRPC cohort [15] were analyzed. Of those, 21 patients (10%) were found to have high (z-score > 1) *FAP* mRNA expression (Figure 1), as a result of gene amplification and copy number gains (Figure 2). There were no structural variants.

**Figure 3:**
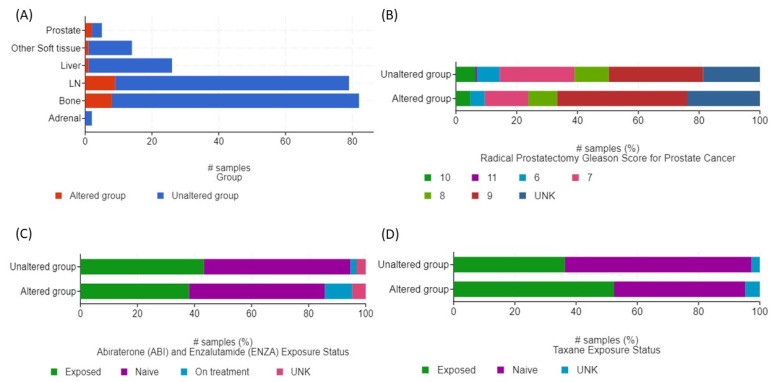
Distribution of mCRPC patients with altered (high) and unaltered tumor *FAP* mRNA expression according to (**A**) tumor biopsy sites, (**B**) Gleason scores at RP, (**C**) abiraterone and enzalutamide exposure status, and (**D**) taxane exposure status. #: number.

The median age at diagnosis was 61 years and the median PSA of all patients was 27.5 (0.68–2000 ng/mL). Other key clinical characteristics including tissue sites of biopsy, RP Gleason score, ARTA (abiraterone or enzalutamide) and taxane exposure status between high and unaltered groups are depicted in Figure 2. The tumor sites studied were bone (*n* = 82) and lymph nodes (*n* = 79), followed by the liver (*n* = 26), other soft tissues (*n* = 9), the prostate (*n* = 5), and the adrenal glands (*n* = 2) (Figure 3A). Patients with high tumor *FAP* expression had proportionally higher Gleason 9 scores at RP (9/21 or 43% vs. 58/187 or 31%) (Figure 3B). Ten out of 21 (48%) patients with high tumoral *FAP* expression were either previously exposed or on-treatment with ARTA (abiraterone or enzalutamide), while 85/187 (45%) had the same exposure in the unaltered group (Figure 3C). A greater proportion of patients with high *FAP* tumor expression had received taxanes prior to biopsy (11/21 or 52%) compared to those with unaltered *FAP* transcript levels (68/187 or 36%) (Figure 3D).

There were no statistically significant differences in the distribution of RP Gleason scores (Chi-squared test *q*-value = 0.948), ARTA exposure (Chi-squared test *q*-value = 0.420) or taxane exposure (Chi-squared test *q*-value = 0.420) between patients with high vs. unaltered *FAP* mRNA expression.

**Figure 4:**
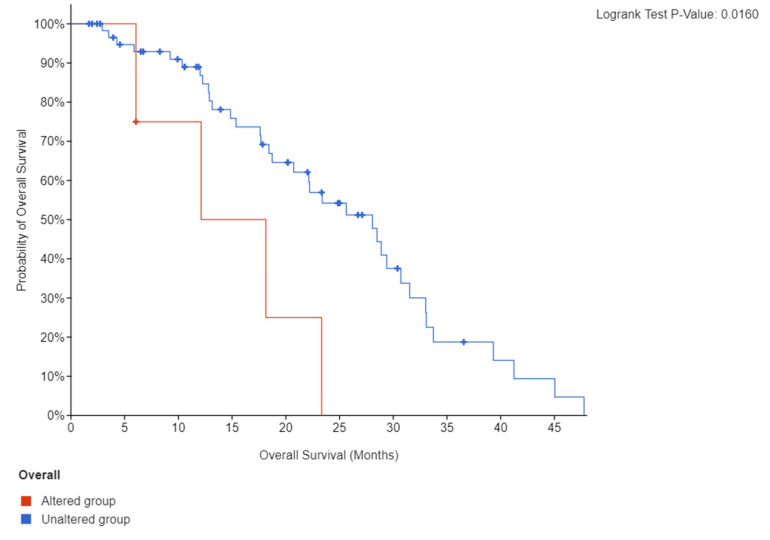
Kaplan-Meier curve of OS according to FAP mRNA expression (high vs. unaltered).

Patients with tumors harboring high *FAP* mRNA expression had a significantly shorter median OS (12 months) compared to those without altered *FAP* expression (28 months, log-rank *p* = 0.016) (Figure 4).

**Figure 5:**
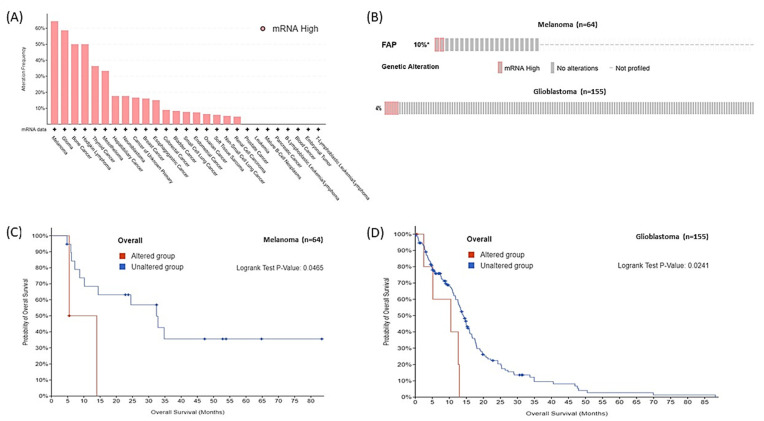
(**A**) Frequency of high *FAP* mRNA in various human cell lines from CCLE. (**B**) Oncoplots of *FAP* mRNA expression in melanoma and glioblastoma patient cohorts. Kaplan Meier curves of OS according to *FAP* mRNA expression (high vs. unaltered) in melanoma (**C**) and glioblastoma (**D**) cohorts. *: % of profiled.

To validate our findings in other tumor types, we assessed *FAP* mRNA expression across 947 human cancer cell lines from the Cancer Cell Line Encyclopedia (CCLE) [16]. The highest transcript levels above a z-score of 1.0 were found in melanoma (64.4% of 59 cell lines) and glioma (58.7% of 46 cell lines) (Figure 5A). We thus sought to examine the prognostic utility of high *FAP* mRNA in two independent cohorts of melanoma (*n* = 64), and glioblastoma (*n* = 155) from The Cancer Atlas Database (TCGA) [17,18]. High *FAP* mRNA was found in 10% and 4% of tumors, respectively, and was associated with shorter median OS (melanoma cohort: 5.5 vs. 32.4 mos, *p* = 0.046; glioblastoma cohort: 10.4 vs. 14 mos, *p* = 0.024) (Figure 5B–D).

**Figure 6:**
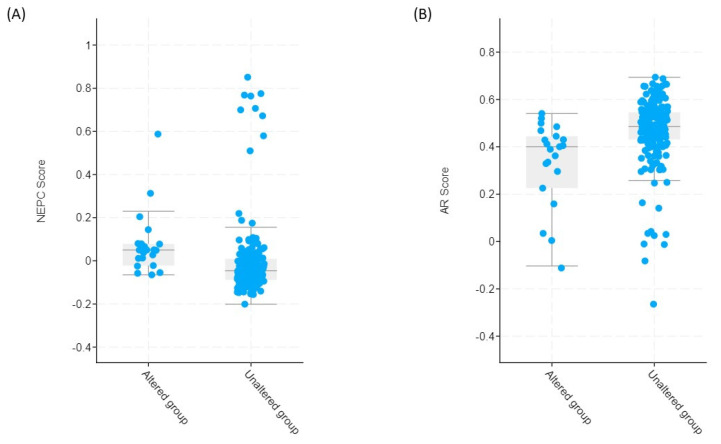
NEPC (**A**) and AR (**B**) scores in mCRPC patients with altered (high) and unaltered tumor FAP mRNA expression.

We then specifically sought to assess whether high *FAP* expression is associated with presence of neuroendocrine features. NEPC and AR signaling scores were assessed in each tumor sample and compared between subgroups with high vs. unaltered *FAP* transcript levels. Tumors with high *FAP* expression were characterized by a significantly higher NEPC score of 0.05 (−0.06–0.23) compared to those without alteration in *FAP* transcript levels [−0.04 (−0.20–0.16); *q* < 0.001] (Figure 6A). Reversely, AR score was significantly lower [0.40 (−0.10–0.54); *q* < 0.001] in tumors with high *FAP* expression (Figure 6B).

## 4. Discussion

FAP is an increasingly recognized CAF marker, and high FAP protein expression in various cancers often indicates an aggressive course. This study assessed the clinical relevance of *FAP* transcriptional expression in patients with mCRPC with respect to prognosis and presence of NE features. We found that high *FAP* expression is a harbinger of shorter OS in mCRPC patients. We validated our findings on the negative prognostic significance of *FAP* in independent cohorts of patients with high *FAP*-expressing tumors based on the CCLE, including melanoma and glioma. Additionally, we showed that CRPC tumors with high *FAP* expression are characterized by a higher NEPC score and lower AR score suggesting an enrichment in NE features.

A gradient of increasing FAP protein expression in prostate tissue microarrays (TMA) from 94 patients at different stages of PC (primary PC, patients undergoing neoadjuvant androgen deprivation therapy, CRPC, and NEPC) was recently reported, indicating a significant rise upon disease progression at the CRPC and NEPC states [19]. Our work complements these findings, by demonstrating that high *FAP* transcript levels are associated with worse prognosis of mCRPC patients. Taking a step further, our findings indicate that this aggressive clinical course of patients with high *FAP*-expressing tumors is associated with an enrichment in NE differentiation signals as indicated by a high NEPC score and low AR score.

It is likely that within this highly heterogenous, multicenter cohort, with variable treatment intervals of ARTA (ABI/ENZA) or/and taxanes, small differences in *FAP* expression are difficult to be detected. On the other hand, while it did not reach statistical significance, there was a numeric enrichment of high GS tumors at RP, particularly GS 9, in the high *FAP*-expressing subset compared to those patients whose tumors did not display *FAP* transcript level alterations.

Our findings have important diagnostic and therapeutic implications. First, the feasibility of detecting FAP for imaging purposes with use of small molecules on optical and single-photon computed tomography was recently shown in vitro and in vivo [20]. Furthermore, PET imaging with a FAP-targeted antibody imaging probe, ^89^Zr-B12 IgG, was successfully evaluated in preclinical PC models, demonstrating high tumor uptake and long-term retention of the probe [21]. Clinical evaluation of another PET-probe, ^68^Ga-FAPI PET/CT demonstrated multiple metastatic lesions confirmed by conventional CT scan in three metastatic CRPC and NEPC patients, supporting the role of FAP as a key diagnostic target [19]. Interestingly, when ^68^Ga-FAPI was compared head-to-head with ^18^F-FDG PET/CT in a multicenter study of 71 patients with various primaries, the former had similar quantitative tumor uptake, but lower background uptake, yielding improved diagnostic information particularly in tumor areas with high physiological ^18^F-FDG uptake [22]. From a therapeutic perspective, targeting of stromal FAP with monoclocal antibodies can be effective, particularly when combined with tumor targeting approaches against the prostate tumor antigen tumor-associated calcium signal transducer 2 (TROP2) using engineered natural killer NK-92 cells expressing CD64 [23].

Collectively, while our analysis requires additional prospective validation, our findings strengthen the clinical value of *FAP* as a surrogate marker of NE differentiation and prognosticator in metastatic CRPC, providing the rational for its diagnostic and therapeutic targeting to improve outcomes of these patients.

## Data Availability

Publicly available data supporting results of this study were previously deposited in GitHub, https://github.com/cBioPortal/datahub/tree/master/public/prad_su2c_2019, accessed on 6 January 2022, and can be accessed through the cBioPortal for Cancer Genomics (www.cbioportal.org, accessed on 6 January 2022).

## References

[1] Chen Y., McAndrews K.M., Kalluri R. (2021). Clinical and therapeutic relevance of cancer-associated fibroblasts. Nat. Rev. Clin. Oncol..

[2] Wang L., Cao L., Wang H., Liu B., Zhang Q., Meng Z., Wu X., Zhou Q., Xu K. (2017). Cancer-associated fibroblasts enhance metastatic potential of lung cancer cells through IL-6/STAT3 signaling pathway. Oncotarget.

[3] Huang M., Fu M., Wang J., Xia C., Zhang H., Xiong Y., He J., Liu J., Liu B., Pan S. (2021). TGF-β1-activated cancer-associated fibroblasts promote breast cancer invasion, metastasis and epithelial-mesenchymal transition by autophagy or overexpression of FAP-α. Biochem. Pharmacol..

[4] Wu Q.Q., Zhao M., Huang G.Z., Zheng Z.N., Chen Y., Zeng W.S., Lv X.Z. (2020). Fibroblast Activation Protein (FAP) Overexpression Induces Epithelial-Mesenchymal Transition (EMT) in Oral Squamous Cell Carcinoma by Down-Regulating Dipeptidyl Peptidase 9 (DPP9). OncoTargets Ther..

[5] Meyer S.N., Galván J.A., Zahnd S., Sokol L., Dawson H., Lugli A., Zlobec I. (2019). Co-expression of cytokeratin and vimentin in colorectal cancer highlights a subset of tumor buds and an atypical cancer-associated stroma. Hum. Pathol..

[6] Liu J., Huang C., Peng C., Xu F., Li Y., Yutaka Y., Xiong B., Yang X. (2018). Stromal fibroblast activation protein α promotes gastric cancer progression via epithelial-mesenchymal transition through Wnt/β-catenin pathway. BMC Cancer.

[7] Zou B., Liu X., Zhang B., Gong Y., Cai C., Li P., Chen J., Xing S., Chen J., Peng S. (2018). The Expression of FAP in Hepatocellular Carcinoma Cells is Induced by Hypoxia and Correlates with Poor Clinical Outcomes. J. Cancer.

[8] Sasaki K., Sugai T., Ishida K., Osakabe M., Amano H., Kimura H., Sakuraba M., Kashiwa K., Kobayashi S. (2018). Analysis of cancer-associated fibroblasts and the epithelial-mesenchymal transition in cutaneous basal cell carcinoma, squamous cell carcinoma, and malignant melanoma. Hum. Pathol..

[9] Guan J., Zhang H., Wen Z., Gu Y., Cheng Y., Sun Y., Zhang T., Jia C., Lu Z., Chen J. (2014). Retinoic acid inhibits pancreatic cancer cell migration and EMT through the downregulation of IL-6 in cancer associated fibroblast cells. Cancer Lett..

[10] Davies A.H., Beltran H., Zoubeidi A. (2018). Cellular plasticity and the neuroendocrine phenotype in prostate cancer. Nat. Rev. Urol..

[11] Vlachostergios P.J., Puca L., Beltran H. (2017). Emerging Variants of Castration-Resistant Prostate Cancer. Curr. Oncol. Rep..

[12] Beltran H., Prandi D., Mosquera J.M., Benelli M., Puca L., Cyrta J., Marotz C., Giannopoulou E., Chakravarthi B.V., Varambally S. (2016). Divergent clonal evolution of castration-resistant neuroendocrine prostate cancer. Nat. Med..

[13] Puca L., Vlachostergios P.J., Beltran H. (2019). Neuroendocrine Differentiation in Prostate Cancer: Emerging Biology, Models, and Therapies. Cold Spring Harb. Perspect. Med..

[14] Vlachostergios P.J., Papandreou C.N. (2015). Targeting neuroendocrine prostate cancer: Molecular and clinical perspectives. Front. Oncol..

[15] Abida W., Cyrta J., Heller G., Prandi D., Armenia J., Coleman I., Cieslik M., Benelli M., Robinson D., Van Allen E.M. (2019). Genomic correlates of clinical outcome in advanced prostate cancer. Proc. Natl. Acad. Sci. USA.

[16] Barretina J., Caponigro G., Stransky N., Venkatesan K., Margolin A.A., Kim S., Wilson C.J., Lehár J., Kryukov G.V., Sonkin D. (2016). The Cancer Cell Line Encyclopedia enables predictive modelling of anticancer drug sensitivity. Nature.

[17] Snyder A., Makarov V., Merghoub T., Yuan J., Zaretsky J.M., Desrichard A., Walsh L.A., Postow M.A., Wong P., Ho T.S. (2014). Genetic basis for clinical response to CTLA-4 blockade in melanoma. N. Engl. J. Med..

[18] Brennan C.W., Verhaak R.G., McKenna A., Campos B., Noushmehr H., Salama S.R., Zheng S., Chakravarty D., Sanborn J.Z., Berman S.H. (2013). The somatic genomic landscape of glioblastoma. Cell.

[19] Kesch C., Yirga L., Dendl K., Handke A., Darr C., Krafft U., Radtke J.P., Tschirdewahn S., Szarvas T., Fazli L. (2021). High fibroblast-activation-protein expression in castration-resistant prostate cancer supports the use of FAPI-molecular theranostics. Eur. J. Nucl. Med. Mol. Imaging.

[20] Slania S.L., Das D., Lisok A., Du Y., Jiang Z., Mease R.C., Rowe S.P., Nimmagadda S., Yang X., Pomper M.G. (2021). Imaging of Fibroblast Activation Protein in Cancer Xenografts Using Novel (4-Quinolinoyl)-glycyl-2-cyanopyrrolidine-Based Small Molecules. J. Med. Chem..

[21] Hintz H.M., Gallant J.P., Vander Griend D.J., Coleman I.M., Nelson P.S., LeBeau A.M. (2020). Imaging Fibroblast Activation Protein α Improves Diagnosis of Metastatic Prostate Cancer with Positron Emission Tomography. Clin. Cancer Res..

[22] Giesel F.L., Kratochwil C., Schlittenhardt J., Dendl K., Eiber M., Staudinger F., Kessler L., Fendler W.P., Lindner T., Koerber S.A. (2021). Head-to-head intra-individual comparison of biodistribution and tumor uptake of 68Ga-FAPI and 18F-FDG PET/CT in cancer patients. Eur. J. Nucl. Med. Mol. Imaging.

[23] Hintz H.M., Snyder K.M., Wu J., Hullsiek R., Dahlvang J.D., Hart G.T., Walcheck B., LeBeau A.M. (2021). Simultaneous Engagement of Tumor and Stroma Targeting Antibodies by Engineered NK-92 Cells Expressing CD64 Controls Prostate Cancer Growth. Cancer Immunol. Res..

